# General Medicine and Therapeutics

**Published:** 1893-03

**Authors:** 


### *SELECTIONS AND ABSTRACTS.*

### GENERAL MEDICINE AND THERAPEUTICS.

Edited by Dr. LUTHER B. GRANDY.

### The Examination of Sputum for Tubercle Bacilli.

The most practical use that has thus far been made of the discovery of the tubercle bacillus is, undoubtedly, its value in the diagnosis of pulmonary tuberculosis. The satisfactory results in the treatment of this disease that are constantly being reported, when the patients are placed under proper conditions in the beginning of the invasion of the bacilli, emphasize the importance of a correct and *early* diagnosis. It is well known that when the changes in the lung tissue have advanced to a sufficient extent to admit of a differentiation by physical signs or symptoms there is little hope of an ultimate recovery. The difficulty that has heretofore existed in recognizing tuberculosis in its incipient stage has compelled many a physician to allow his patients to pass beyond the reach of medical aid before the real nature of their disease was determined and an opportunity given them to test the virtue of a better climate or other curative agents which are proving so beneficial when applied at the proper time. It is to avoid this error that physicians should avail themselves of all the assistance that scientific research has developed for them.

From among a large number of methods that have been published the one recommended by Dr. Gabbett appears to us the most practicable, especially for sputum examination.

The sputum (the first expectorated in the morning is preferable) is spread in a very thin layer over the surface of a cover-glass and allowed to dry in the air. The cover-glass preparation (as it is now called) is passed, film upwards, three times through the flame of a spirit lamp or a Bunsen burner, in order to fix the film on the cover-glass so that it will not be lost during the subsequent treatment. It is then covered with a few drops of the staining fluid (which consists of fuchsin 1 gram; absolute alcohol 10 cubic centimeters; 5 per cent. carbolic acid 100 cubic centimeters). The stain is allowed to act for about two minutes. The preparation is then rinsed in water and the film again covered with the decolorizing and counter-stain solution (methylin blue 2 grains; 25 per cent. sulphuric acid 100 cubic centimeters), which is allowed to act from one to two minutes when it is again rinsed in water. It can be mounted in water and examined at once, or allowed to dry when it can be permanently mounted in balsam.

The tubercle bacilli should appear upon a microscopical examination as slender, more or less curved, rod-shaped bodies of a deep red color, the surrounding tissue cells and other bacteria having a blue color. For these solutions care must be taken to secure pure fuchsin and methyline-blue.

In the beginning of the invasion of the bacilli it is often impossible to detect their presence in cover-glass preparations made from a single specimen of sputum. As a large number of negative results do not positively affirm the absence of the disease, several specimens should be examined.

Biedert (*Berl. Klin. Wochenschrift*, 1886, No. 42, p. 43, ibid 1891, No. 2, p. 31) has recommended a very excellent method for detecting the bacilli when they are present in very small numbers, which in the hands of a careful workman is much simpler than the repeated examination of cover-glass preparations made directly from the sputum. The method consists in mixing a considerable quantity of sputum with twice its volume of a 2 per cent. solution of caustic soda and boiling the mixture until it is perfectly fluid, after which it is placed in a conical dish and allowed to stand until a sediment is formed. The soda solution dissolves the tissue cells leaving the bacteria and elastic fibres, if any are present, to form deposits in the bottom of the glass. Upon decanting the supernatant liquid tubercle bacilli, otherwise difficult or impossible to find, can readily be discovered in properly stained cover-glass preparations made from the sediment. Although this process requires considerable time the advantages to be gained are obvious, and the importance of an early diagnosis overbalances the time and care that any of the methods require to detect the presence of the invading bacteria.—*Med. and Surg. Reporter.*

---

### THE SPECIFIC TREATMENT OF ENTERIC FEVER.

The form of specific treatment referred to embraces the use of calomel in connection with the tincture of iodine and carbolic acid. This treatment was described by Dr. James C. Wilson, of Philadelphia, nine years ago, and previously recommended by Professor Bartholow.

It is my practice, in commencing the treatment of a case of enteric fever, to direct ten grains of calomel every other day until four doses have been taken. Also a mixture containing one drachm of carbolic acid and a sufficient quantity of tincture of iodine to make four drachms; of this give the patient four drops in a wine-glass of water every four hours.

To one who has been taught that cathartics should always be avoided in the treatment of typhoid fever—and who has not?—the proposal to give a patient suffering with this disease ten grains of calomel, and to repeat this dose at intervals of forty-eight hours for a week, may seem reckless—perhaps even murderous. But this medication does not produce the disastrous results which might be expected by one who believes that he must never give a cathartic in typhoid fever. I must admit that when I began to use calomel in this way I prescribed smaller doses, and subsequently inquired in regard to their effect with trepidation. But observing that unpleasant effects seldom, and dangerous effects never, followed their use, and that, furthermore, these smaller doses sometimes failed to produce catharsis, I increased the dose to ten grains. It will generally be found that unpleasant effects are no more likely to follow a dose of this size than a smaller one. It is, moreover, desirable to give a large enough dose to insure a movement of the bowels, on account of the danger of salivation when the drug is retained in the body longer than twenty-four hours.

An experience of eight years in the use of this treatment, in such cases of enteric fever as have fallen under my observation in a moderately extensive general practice, has convinced me that its value is greatly underestimated by most physicians. I am confident that by this treatment the duration of enteric fever may be shortened, that in some cases the course of the disease may be arrested, that its severity may be mitigated, and that, in most cases, dangerous complications may be avoided.—*Dr. Gustavus Eliot, in Coll. and Clinical Record.*

---

### WHAT THE PUBLIC SHOULD KNOW ABOUT SCARLATINA.

Scarlet fever (called also scarlet rash, canker rash, malignant sore throat, etc.) is one of the infectious diseases distinguished by its peculiar rash or "eruption." The essential facts of this disease needful to be known for its prevention or restriction are:

*First.*—That its special infection is developed in the eruption in the skin, and given off thence to persons and clothing, and to bedding and other things in occupied rooms, and that it continues to be given off constantly till the skin is entirely well and all scruff or scruffiness has entirely disappeared.

*Second.*—That the slightest attack of the disease in one child may provoke a severe attack in another exposed to it, and that when it is epidemic a large proportion of the cases are often of so slight a type as not to be recognizable at first, or, often, till the affection has been given to others.

*Third.*—The period of "incubation" (time from taking the infection till the first symptoms appear) is rarely longer than seven days (one week), sometimes but a few hours.

*Fourth.*—The patient is infectious, and so dangerous to others, for at least forty days, and as a rule for seven or eight weeks, *i. e.*, till the skin is entirely well and free of scruff, particularly on the palms of the hands and soles of the feet.

*Fifth.*—The infection preserves its virulence in clothing and bedding hung up in closets or packed in trunks, clothing of the patient or his attendant or others exposed to him. So stored, the infection clings to such things sometimes for months, ready when the article is shaken in the air to float off and infect the nearest child.

*Sixth.*—Boiling water or a strong solution of quicklime will destroy this infection in clothing or bedding, and the last will do the same for floors and walls if freely applied. On the person of the sick a simple ointment (one part mutton tallow, two parts fresh lard melted separately and mixed thoroughly till cold) is the best and easiest means of limiting the spread of the infection. Used all over the body, it does two things—relieves the fever and itching of the rash, and confines the loose particles of skin containing virus to its surface or to the body, clothing or the sheets of the bed, thus preventing its escape into the air.—*Minnesota State Board of Health.*

---

### GUM-LANCING.

In a letter to the *University Medical Magazine*, on the subject of lancing the gums of teething children, Dr. H. C. Wood says as follows:

Clinically, I am absolutely sure that I have seen convulsions, sick stomach, great restlessness, fever, and various other functional disturbances in young children, immediately cured by the use of the gum-lancet, after the failure of various other well-directed measures for relief. Theoretically, I am in accord with Dr. Kirk, in believing that Dr. Forchheimer absolutely misses the point of the matter by his failure to understand that the good achieved is not due to the local blood letting or to the relief of the inflammation of the gum, but to the removal of the backward pressure upon an extraordinarily sensitive and, at such times, congested nerve pulp. As was long ago pointed out by Dr. J. W. White, at the period of eruption the roots of the teeth are yet incomplete. "Instead of the conical termination and minute foramen, which characterizes a perfected tooth, the aperture is nearly as large as the root itself, and thus when the sensitive pulp, composed of connective tissue, blood-vessels and nerves, is in a condition of irritation because of the morbid activity of the process of dentition—augmented vascular and nervous action—there may be produced a hyperemia sufficient possibly to cause the protrusion of a part of the mass from the incomplete aperture of the root, giving abundant cause for extreme constitutional disturbance."

I have myself seen a seemingly incurable epilepsy in an adult permanently cured by the removal of a persistent milk or first dentition tooth. Amaurosis and various other conditions in the adult are well known to be the result of irritation of the trigeminal nerve by faulty teeth. How much more evil is to be expected from the teeth irritation in the child!

In conclusion, I reaffirm that whatever the theory in the matter may be, I am positive that gum-lancing is a most important therapeutic measure. It is essential, however, that it should be thorough, and with the object of dividing the dense tissues that bind down the teeth.—*Medical World.*

---

### ADMINISTRATION OF PODOPHYLLIN.

When it is desirable to prescribe podophyllin, it is well to remember its solubility in tincture of ginger, in which it may be administered as follows:

℞. Resinæ podophylli, gr. ij.
Tinct. zingiberis, f℥j.

M. SIG.—Dose, a teaspoonful in a glass of sweetened water on retiring.—*Med. and Surg. Reporter.*

---

### SUMMER DIARRHŒA.

For children one year of age:

℞. Zinci sulphocarbolat., gr. v.
Bismuth subnitr., gr. xv.
Pepsin saccharat, ʒ ss.
M. Et. in chart. No. xv. div.

Sig.—One powder every hour until stools become inodorous; then every two to four hours.—*Waugh, Times and Register.*

